# Cell-penetrating peptide-driven Cre recombination in porcine primary cells and generation of marker-free pigs

**DOI:** 10.1371/journal.pone.0190690

**Published:** 2018-01-09

**Authors:** Qianqian Kang, Zhaolin Sun, Zhiyuan Zou, Ming Wang, Qiuyan Li, Xiaoxiang Hu, Ning Li

**Affiliations:** State Key Laboratories for Agrobiotechnology, College of Biological Sciences, China Agricultural University, Beijing, China; Friedrich-Loeffler-Institute, GERMANY

## Abstract

Cell-penetrating peptides (CPPs) have been increasingly used to deliver various molecules, both *in vitro* and *in vivo*. However, there are no reports of CPPs being used in porcine fetal fibroblasts (PFFs). The increased use of transgenic pigs for basic research and biomedical applications depends on the availability of technologies for efficient genetic-modification of PFFs. Here, we report that three CPPs (CPP5, TAT, and R9) can efficiently deliver active Cre recombinase protein into PFFs via an energy-dependent endocytosis pathway. The three CPP**–**Cre proteins can enter PFFs and subsequently perform recombination with different efficiencies. The recombination efficacy of CPP5**–**Cre was found to be nearly 90%. The rate-limiting step for CPP**–**Cre-mediated recombination was the step of endosome escape. HA2 and chloroquine were found to improve the recombination efficiency of TAT**–**Cre. Furthermore, we successfully obtained marker-free transgenic pigs using TAT**–**Cre and CPP5**–**Cre. We provide a framework for the development of CPP-based farm animal transgenic technologies that would be beneficial to agriculture and biomedicine.

## Introduction

Transgenic technology is important for biomedical and agricultural applications. Traditional transgenic methods include microinjection, electroporation, nucleofection, and the use of liposomes and viral vectors [1–5]. However, these methods can be inefficient and time consuming, and are associated with cytotoxicity. Recently, “cell-penetrating peptide technology” has been reported to be an efficient and safe approach for gene delivery.

Cell-penetrating peptides (CPPs), also known as protein transduction domain (PTD) peptides, can be used to deliver a large variety of molecules, including drugs, nucleic acids, peptides, and large proteins, into mammalian cells, both *in vitro* and *in vivo*, with no apparent toxicity [6–10]. TAT (YGRKKRRQRRR) [11], R9 (RRRRRRRRR) [12], and CPP5 (KLVPM) [13] are three widely investigated and frequently used CPPs. Although the transduction mechanism of CPPs is not fully understood, it is clear that two types of cellular uptake mechanisms exist [14–16], namely an endocytosis-dependent mechanism [17] and a direct membrane translocation mechanism [18]. Understanding the transduction mechanism will further improve the efficacy of CPPs in basic research and in potential therapeutic applications. The cell permeable transduction technology has been successfully applied to various mammalian cells, such as fibroblasts [19], hematopoietic stem cells (HSCs) [20], and mouse and human embryonic stem cells [21,22]. Moreover, CPPs was demonstrated to deliver “cargo” to immature wheat embryos and plants tissues [23,24]. However, there are no reports on the use of CPPs in porcine fetal fibroblasts and in the generation of transgenic pigs.

Transgenic domestic animals have important applications in agriculture and biomedical research [25,26]. Pigs are considered to be more suitable biomedical models than rodents for studying human diseases because they are similar to humans in their physiology and anatomy, and have similar lifespan [27]. It is becoming one of the most important sources of donor organs for xenotransplantation [28,29]. In the process of generating transgenic pigs, antibiotic-resistance genes are required to select transgenic cells as donor cells for successful somatic cell nuclear transfer (SCNT), which is the most effective method for producing transgenic farm animals [30–32]. The presence of antibiotic-resistance genes in transgenic animals can have side effects: firstly, it might cause changes in the regulation of adjacent genes and might attenuate the expression of the gene of interest [33,34]; secondly, it restricts multiple genetic manipulations in the same host [35]; thirdly, it might arouse public concern about biological safety. It has been reported that the Cre/loxP system can be used to remove selective marker genes in cells of many species [36,37]. Using a cell-permeable Cre protein can induce recombination in nearly 100% of human embryonic stem (hES) cells, without any apparent toxicity [22]. However, the removal of selection marker genes has been poorly investigated in transgenic pigs. It has recently been reported that marker-free transgenic pigs can be obtained using the “Sleeping Beauty transposon system” and the Cre/loxP system [38–40]; however, the Sleeping Beauty transposon system is labor intensive and requires screening for single-copy marker gene integration and breeding. The use of the Cre plasmid or mRNA also requires additional electroporation process and has the risk of unwanted integrations with the use of a Cre expression plasmid. Thus, it is necessary to establish a simple, efficient, and reliable method to remove the selection marker genes.

In this study, we evaluated the actual potency and mechanism of three prominent CPPs, namely TAT, R9, and CPP5, in promoting the translocation of Cre across the plasma membrane of PFFs and in subsequent recombination. We successfully generated marker-free transgenic pigs using these methods. We expect that this method will serve as a powerful tool for the rapid and efficient genetic manipulation of PFFs and will accelerate the development of transgenic pigs for agricultural and biomedical applications.

## Materials and methods

### Ethics statement

All the animal work in this study was approved by the Institutional Animal Care and Use Committee of the China Agricultural University with approved number SKLAB-2012-04-05. All the procedures were performed in strict accordance with the Guide for the Care and Use of Laboratory Animals. We performed all surgeries under sodium pentobarbital anesthesia and tried our best to minimize animals suffering.

### Plasmid construction

The pTAT-Cre vector was purchased from Addgene (Plasmid #35619). The pCPP5 and R9 sequences were generated by the single-strand oligo annealing method using primers CPP5-R, CPP5-F, R9-R and R9-F (S1 Table). The CPP5 and R9 sequences were then inserted into the pTAT-Cre vector at the *Nco*I and *Nde*I restriction sites, respectively, and the constructs were named pCPP5-Cre and pR9-Cre, respectively. The pCAGSTOP2 vector was digested with *Bam*HI and *Sac*I, and the three PCR-amplified fragments (mCherry from pmCherry-N1, *Neo* from pACN, and EGFP from pEGFP-N1) using appropriate primers (S1 Table) were fused together by the In-Fusion method (Clontech, Dalian, China, Code: 639648). The resulting plasmid was named pDFR and was used as the substrate to assay protein activity *in vitro*.

### Prokaryotic expression and purification of CPPs-Cre recombinase

All the CPPs-Cre prokaryotic expression vectors were transformed into BL21 Escherichia coli. Cultured the bacteria for 2.5 h at 37°C until the OD600 of medium reach to 0.5, and then induced with 0.2 mM IPTG at 16°C overnight. Cells were harvested and then lysed in lysis buffer (10 mM NaH_2_PO_4_, 10 mM K_2_HPO_4_, 300 mM NaCl, 10 mM 2-mercaptoethanol and 0.1 mM PMSF). After centrifuging the lysate, the cleared lysate was loaded onto Ni-NTA column and then G25 column to purify and elute the CPPs-Cre protein in DMEM (Gibco, New York, NY, USA) with 10% glycerol (w/v). The amount of protein was quantified using the Bradford assay (Bio- Rad).

### Isolation and culture of PFFs

Porcine fetal fibroblasts were isolated from the fetuses of transgenic or wild-type pigs and cultured in DMEM (Gibco) supplemented with 10% fetal bovine serum (FBS; Gibco, New York, NY, USA), 100 units/ml penicillin and 100 μg/ml streptomycin (Gibco).

### Fluorescently labeled CPPs-Cre proteins actively associate with PFFs

Porcine fetal fibroblasts were seeded overnight onto 96-well plates at a density of 5,000 cells/well and were washed and incubated in serum-free medium for 3 h at 37°C. The cells were then treated with CPPs-Cre-Alexa Fluor488 (Alexa Fluor 488 Protein Labeling Kit, Molecular Probes, Catalogue: A-10235) proteins for 3 h in serum-free medium at 37°C or 4°C as indicated. They were then placed on ice, washed several times with PBS, trypsinized, and placed on ice for FACS. The cell association was quantitated by the moFLo XDP (BECKMAN COULTER) flow cytometer equipped with analysis software (Summit 5.2). The live cell population was gated by forward scatter/side scatter, and 1, 000–2,000 live cells were used for each analysis.

### MTT assay used to detect the cytotoxicity

About 5,000 PFFs were plated on 96-well tissue culture plate and cultivated for 24h. Then the commercial MTT assay kit (Beyotime Biotechnology, Beijing, china, Code: C0009) were used to investigate the cytotoxicity of different concentration of CPPs-Cre proteins and the cytotoxicity of HA2 and chloroquine in PFFs in different concentration at 37°C for 3h.

### The colocalization of CPPs-Cre and macropinosomes in porcine fetal fibroblast

To visualize CPPs-Cre internalization, the CPPs-Cre were fluorescently labeled with Alexa 488 (Molecular Probes). The PFFs were incubated with 2 μM fluorescent CPPs-Cre-488 and 4 μM FM4-64 (Millipore, Catalogue: 574799) for 3 h, and then were washed by PBS for twice and cultured in DMEM (Gibco) with 10% FBS (Gibco). The images were acquired using a Nikon confocal microscope.

### Recombination efficiency of CPPs-Cre estimated by standard curve analysis method

Standard curve analysis was carried out according to Lee et al.^39^. Briefly, the cloned PCR product (600 bp) corresponding to marker-gene cloned into pMD19-T vector (2.7 kb, TaKaRa, Dalian, China, TaKaRa Code: 6013), and the constructed plasmid (0.11pg, 3.3kb) was mixed with WT pig genomic DNA (100 ng, 3.03 Gbp) such that the mixture contained one marker-gene clone per haploid genome. The WT genomic DNA was assumed as 100% recombination efficiency, therefore, the mixture was serially diluted in the solution containing WT pig genomic DNA to generate a standard curve stood for different recombination efficiency. Then analyzed the sample and the serial mixture by PCR using the primers P1 and P2 (S1 Table) and calculate the recombination efficiency of each sample using the software Bandscan 5.0.

### TAT-Cre protein transduction and PCR identification the marker-free cell clones and somatic cell nuclear transfer (SCNT)

TAT-Cre recombinase proteins were introduced into cells by adding to the culture medium from pig heterozygous myostatin gene knockout (*MSTN*^+/-^) PFFs, which were generated by our lab in a previous study on myostatin gene knockout pigs. Approximately 1.0×10^5^ *MSTN*^+/-^ PFFs were plated on 6-well tissue culture plates and cultivated. After 24 h, the cells were then treated with 2 μM TAT-Cre recombinase proteins in DMEM (Gibco) without 10% FBS (Gibco) for 2 h and were then washed and cultivated for another 48 h with 10% FBS (Gibco). Then, the cells were trypsinized and reseeded at a density of approximately 100 cells/dish in medium containing no G418 (Sigma) for 6–8 days. Well-separated colonies were isolated and transferred to 96-well plates, the sub-confluent colonies were then selected, and half of them were used for DNA extraction (QIAGEN DNeasy blood and tissue kit, QIAGEN, Germany, Catalogue: 69506) and PCR identification. PCR was performed for 35 cycles at 94°C for 30 s, 60°C for 30 s, and 72°C for 30 s, then held at 72°C for 10 min, using the primers M-1and M-2 (S1 Table). The other half of the cells was frozen for later use in somatic cell nuclear transfer (SCNT). The positive colonies were then used in SCNT as previously described[41,42].

### PCR and DNA sequencing identification the marker-free piglets

To detect marker-free piglets, genomic DNA was obtained from ear tissue using phenol-chloroform extraction. Genomic DNA (100 ng per reaction) was subjected to PCR analysis using LA-Taq DNA polymerase (TaKaRa, Dalian, China, TaKaRa Code: RR52A) and appropriate primers (S1 Table). PCR products were analyzed by agarose gel electrophoresis. For sequencing analysis, PCR products corresponding to marker-free fragments were purified using QIAquick Gel Extraction Kit (QIAGEN, Germany, Catalogue: 28704) and cloned into the pMD-19T (TaKaRa, Dalian, China, TaKaRa Code: 6013). The cloned plasmids were then sequenced using M13 primers.

### The source of the pigs used in the study

The experiment pigs in my study were commercial breeds Duroc which are feeding at the experimental pig farm of China Agricultural University.

### The method for obtaining porcine fetal fibroblasts

To isolate the porcine fetal fibroblasts, the Duroc sows were slaughtered at 28–30 days after fertilization and the uterus containing the embryos were excised. Fibroblasts were isolated from this preparation via trypsin digestion, placed in DMEM medium with 10% FBS (Gibco), and grown at 37°C.

### The methods used to generate marker-free piglets

TAT-Cre recombinase proteins were introduced into cells by adding to the culture medium from pig heterozygous myostatin gene knockout (*MSTN*^+/-^) PFFs, which were generated by our lab in a previous study on myostatin gene knockout pigs. Approximately 1.0×10^5^ *MSTN*^+/-^ PFFs were plated on 6-well tissue culture plates and cultivated. After 24 h, the cells were then treated with 2 μM TAT-Cre recombinase proteins in DMEM (Gibco) without 10% FBS (Gibco) for 2 h and were then washed and cultivated for another 48 h with 10% FBS (Gibco). Then, the cells were trypsinized and reseeded at a density of approximately 100 cells/dish in medium containing no G418 (Sigma) for 6–8 days. Well-separated colonies were isolated and transferred to 96-well plates, the sub-confluent colonies were then selected, and half of them were used for DNA extraction (QIAGEN DNeasy blood and tissue kit, QIAGEN, Germany, Catalogue: 69506) and PCR identification. PCR was performed for 35 cycles at 94°C for 30 s, 60°C for 30 s, and 72°C for 30 s, then held at 72°C for 10 min, using the primers M-1and M-2 (S1 Table). The other half of the cells was frozen for later use in somatic cell nuclear transfer (SCNT). The positive colonies were then used in SCNT as previously described^59,60^. Briefly, the positive transgenic cells were transferred to enucleated oocytes to produce reconstructed embryos, which were then fused and activated simultaneously by application of two direct current pulses of 1.6 kV/cm for 100 ms each at an interval of 1 s using a BTX 2001 Electro Cell Manipulator (BTX, Inc., San Diego, CA, USA) in activation medium (0.3 M mannitol supplemented with 0.05 mM CaCl2, 0.1 mM MgCl_2_, and 0.01% polyvinyl alcohol in H_2_O). After chemical activation with 2.5 mg/mL cytochalasin B and 10 mg/mL cycloheximide in porcine zygote medium-3, day-2 blastocysts were transferred to synchronous recipient sows with hundreds of embryos per recipient.

### The fate of the animals used in the study

The animals are normal live and used to breed.

## Results

### Expression, purification, and in vitro activity of CPP–Cre fusion proteins

**Fig 1:**
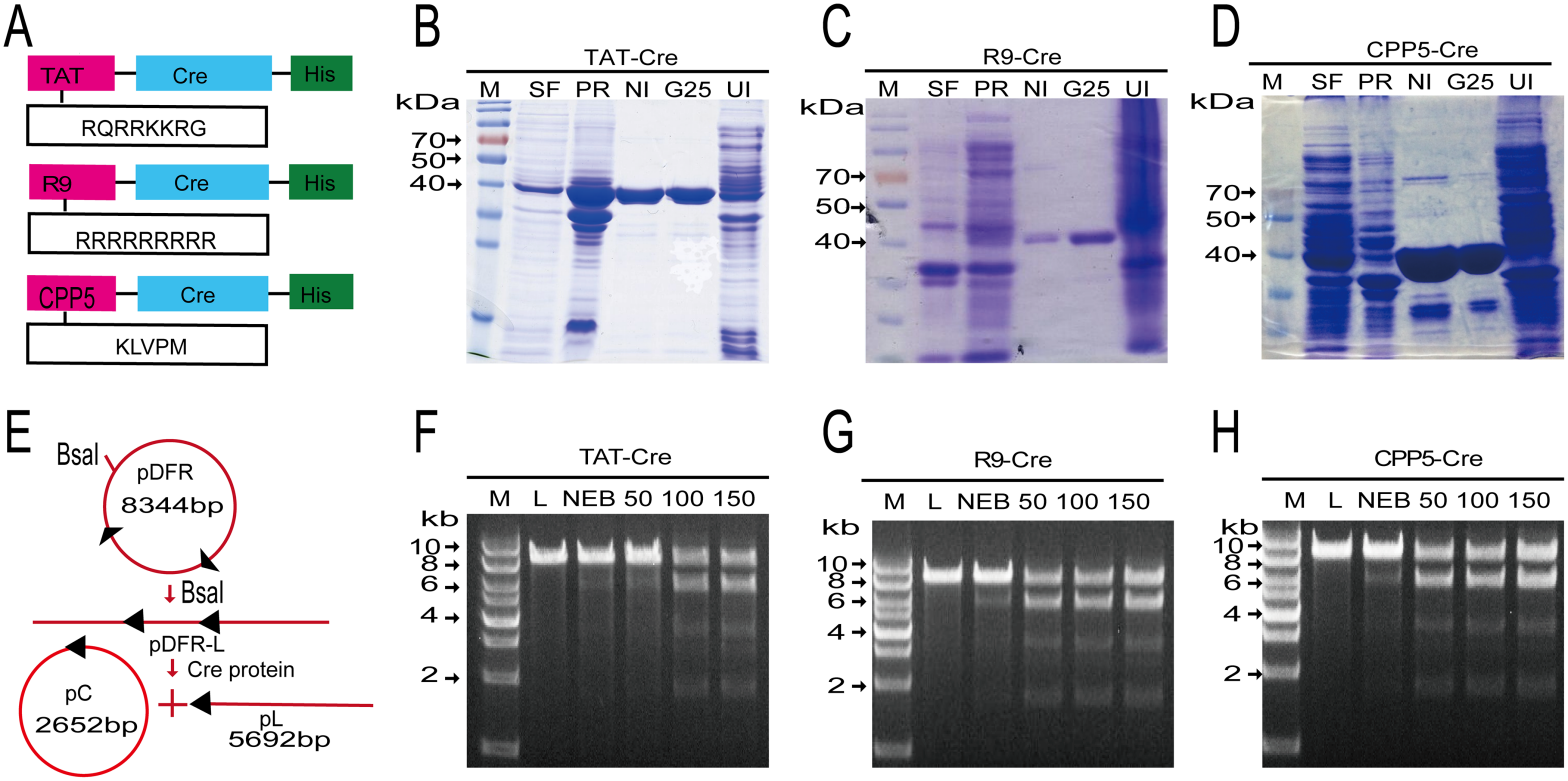
Design of expression cassettes, purification of the three CPP–Cre proteins, and assessment of their activities in an *in vitro* assay. (A) Schematic description of the three CPPs**–**Cre expression constructs. All the constructs encode Cre recombinase with a His-tag (represented by blue and green boxes, respectively). Red boxes represent CPPs (RQRRKKRG): R9 (RRRRRRRRR), TAT (YGRKKRRQRRR), and CPP5 (KLVPM). (B-D) SDS-PAGE analysis of the purification of CPP**–**Cre proteins. M, marker; SF, supernatant fraction; PR, precipitation; Ni, Nickel column; G25, G25 column. (E) Schematic of recombination *in vitro*. The assay substrate, pDFR, was linearized by digestion with *Bsa*I and was used to assess the recombinase activity of the purified Cre proteins. (F-H) Activity of CPP**–**Cre proteins *in vitro*. Reactions were carried out in a 50-μL volume with 300 ng of pDFR and New England Biolabs Cre buffer. Different amounts of purified Cre were added, and the mixtures were incubated at 37°C for 30 min. The reactions were then split in half and resolved on a 1% agarose gel. Lane 1 kb ladder; lane 2, NEB Cre; lane 3, 50 ng Cre; lane 4, 100 ng Cre; lane 5, 150 ng Cre. Note the appearance of four bands including the recombined circular plasmid (migrating at the 2.6 kb pC), the nicked recombined circular plasmid pC-N, the rejoined 5.7 kb “stuffer fragment” pL, and the 8.3kb pDFR-L.

To assess the potency of three CPPs (TAT, R9, and CPP5) in promoting the cellular uptake of Cre recombinase by porcine fetal fibroblasts, we used three expression vectors, pTAT-Cre, pR9-Cre, and pCPP5-Cre (Fig 1A). Soluble proteins were purified from a bacterial host by a two-step method (Fig 1B, 1C and 1D) and were subsequently used to analyze the activity in an *in vitro* recombination reaction. We constructed the plasmid pDFR (8.3 kb), which was used as a substrate (Fig 1E). Incubation of linearized pDFR with Cre resulted in a linearized pDFR-L (5.7 kb) and a recircularized pDFR-C (2.6 kb) (Fig 1E, 1F, 1G and 1H). The *in vitro* assay demonstrated that all the three fusion proteins functioned to recombine the substrate (linearized pDFR, 8.3 kb in size) to generate two bands (2.6 kb and 5.7 kb in size).

### Evaluation of the cell association activities and toxicities of CPP–Cre fusion proteins in PFFs

**Fig 2:**
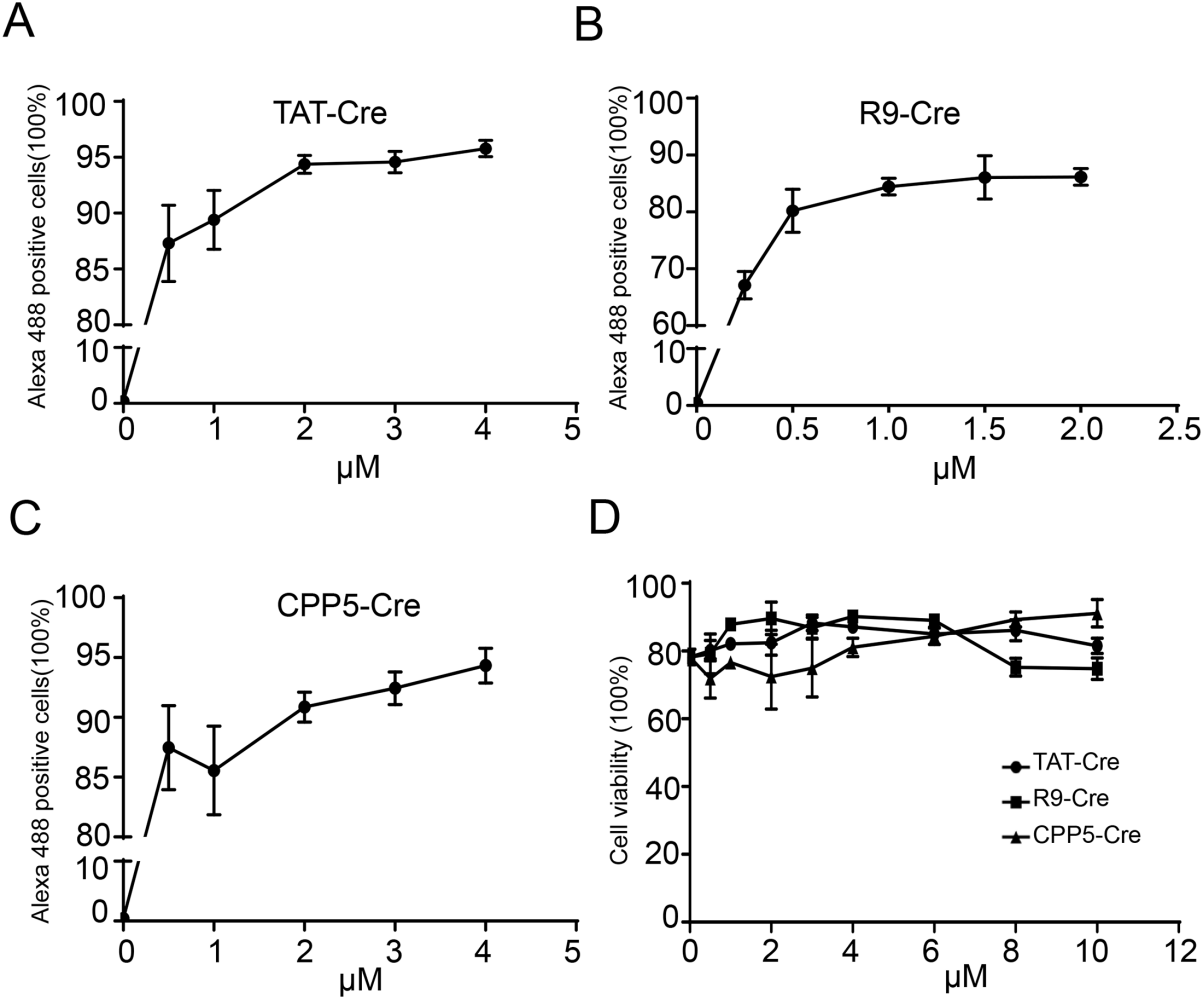
Cell association activities and toxicity of CPP–Cre proteins in porcine fetal fibroblasts (PFFs). (A-C) Cell association activities of the three CPP**–**Cre proteins in PFFs. Flow cytometry analysis of cells treated for 3 h at 37°C at the indicated concentrations of CPP**–**Cre proteins, labeled with Alexa Fluor 488. The cells were washed thoroughly with PBS and then trypsinized to remove the extracellular CPP fusion proteins. Vehicle control (PBS and DMEM) was taken analyze the percentage of Alexa Flor 488 positive. (D) Percentage of living cells among PFFs treated with different concentrations of CPP**–**Cre proteins. Error bars, s.d. (n = 3).

Flow cytometry was used to study the cell association activities of the CPP**–**Cre fusion proteins in porcine fetal fibroblasts. PFFs were incubated with different concentrations of CPP**–**Cre, fluorescently labeled with Alexa Fluor 488, for 3 h at 37°C. The cell association activities of the three recombinant proteins increased with the increasing protein concentration (Fig 2A, 2B and 2C). Interestingly, the cell association activities of TAT**–**Cre and R9**–**Cre increased, and then decreased after the concentrations were increased to 1 and 2 μM, respectively (Fig 2A and 2B), whereas the cell association activity of CPP5**–**Cre continued to increase and did not show a decrease even at 4 μM (Fig 2C). We also used Cre as a negative control and the cell association activities were found to be lower than in the case of TAT**–**Cre (S1 and S2 Figs). The cytotoxicity of the three recombinant proteins was investigated in PFFs using the MTT (3-(4, 5-cimethylthiazol-2-yl)-2, 5-diphenyl tetrazolium bromide) assay. None of these proteins showed significant cytotoxic effects, even at a concentration of 10 μM in the cell culture medium (Fig 2D).

### CPP–Cre enters the PFFs by energy-dependent endocytosis

**Fig 3:**
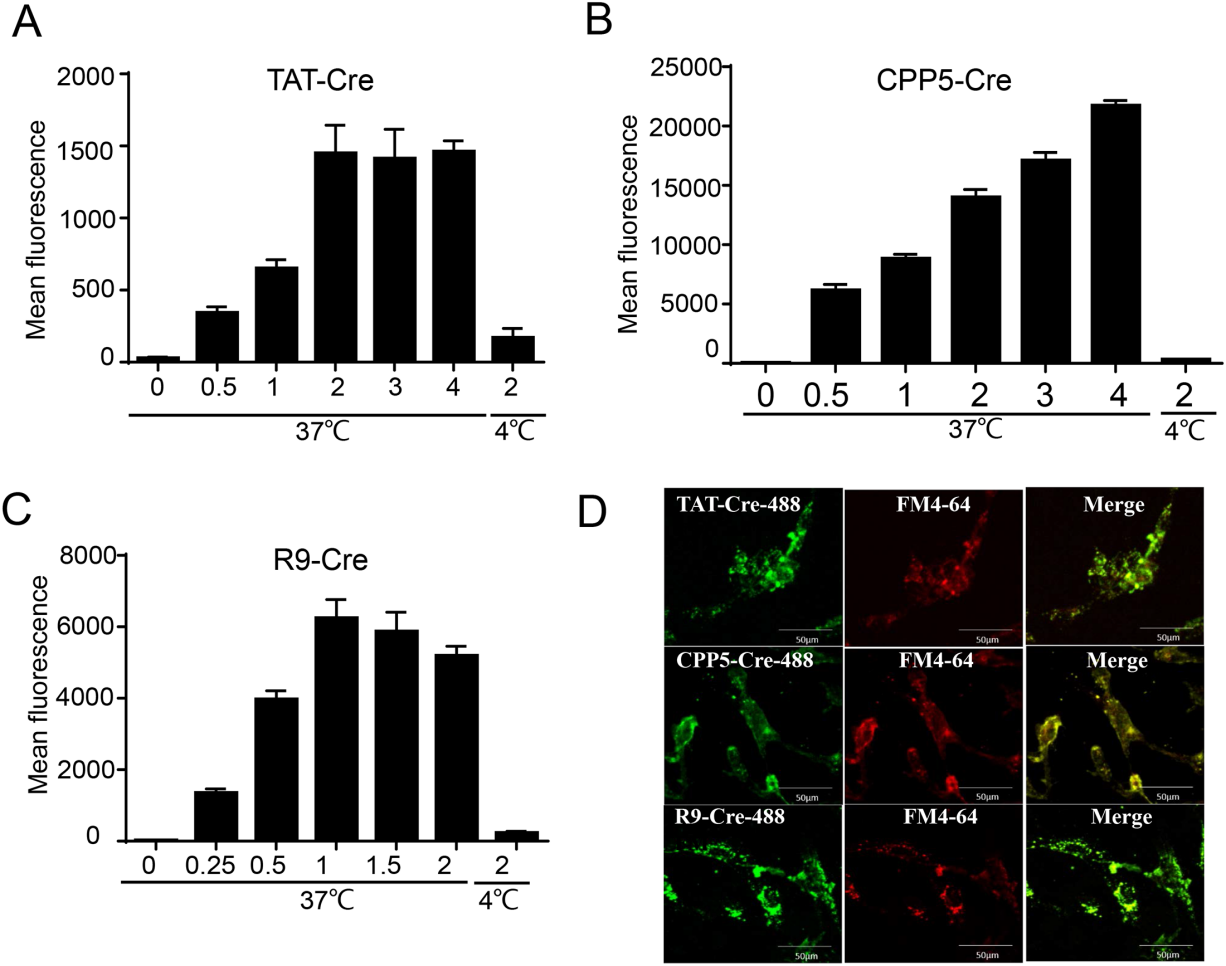
CPP-mediated Cre recombinase transduction efficiency at 4°C and 37°C, and localization of the CPP–Cre proteins. (A-C) Transduction efficiencies of CPP**–**Cre proteins at 4°C and 37°C. Flow cytometry analysis of cells treated for 3 h at 37°C and 4°C at the indicated concentrations of CPP**–**Cre labeled with Alexa Fluor 488. The cells were washed thoroughly with PBS and then trypsinized to remove the extracellular CPP fusion proteins. (D) Confocal images showing endosomal colocalization of CPP**–**Cre-488 proteins and FM4-64 (fluorescent, general endosomal marker) in live cells. Scale bar, 10 μm.

To investigate the mechanism of internalization of the three Cre recombinase proteins into the PFFs, we incubated the PFFs with different concentrations of fluorescein-labeled Cre recombinant proteins for 1 h at 37°C and with 2 μM fluorescein-labeled Cre recombinase for 1 h at 4°C (Fig 3A, 3B and 3C). The protein uptake was inhibited at 4°C for all three recombinant proteins, indicating that transduction of the proteins is energy-dependent in PFFs (Fig 3A, 3B and 3C). We found that fluorescently labeled recombinant proteins (CPP**–**Cre-488) colocalized with a fluorescent endocytosis marker, FM4-64, to intracellular endocytic vesicles in live cells (Fig 3D), as reported in a previous study where the uptake of the TAT-fusion protein was reported to occur by endocytosis in HeLa cells [17]. Our results demonstrated that the three Cre recombinant proteins invaded PFFs by energy-dependent endocytosis.

### Identification of the recombination efficiency of CPP–Cre fusion proteins in PFFs

**Fig 4:**
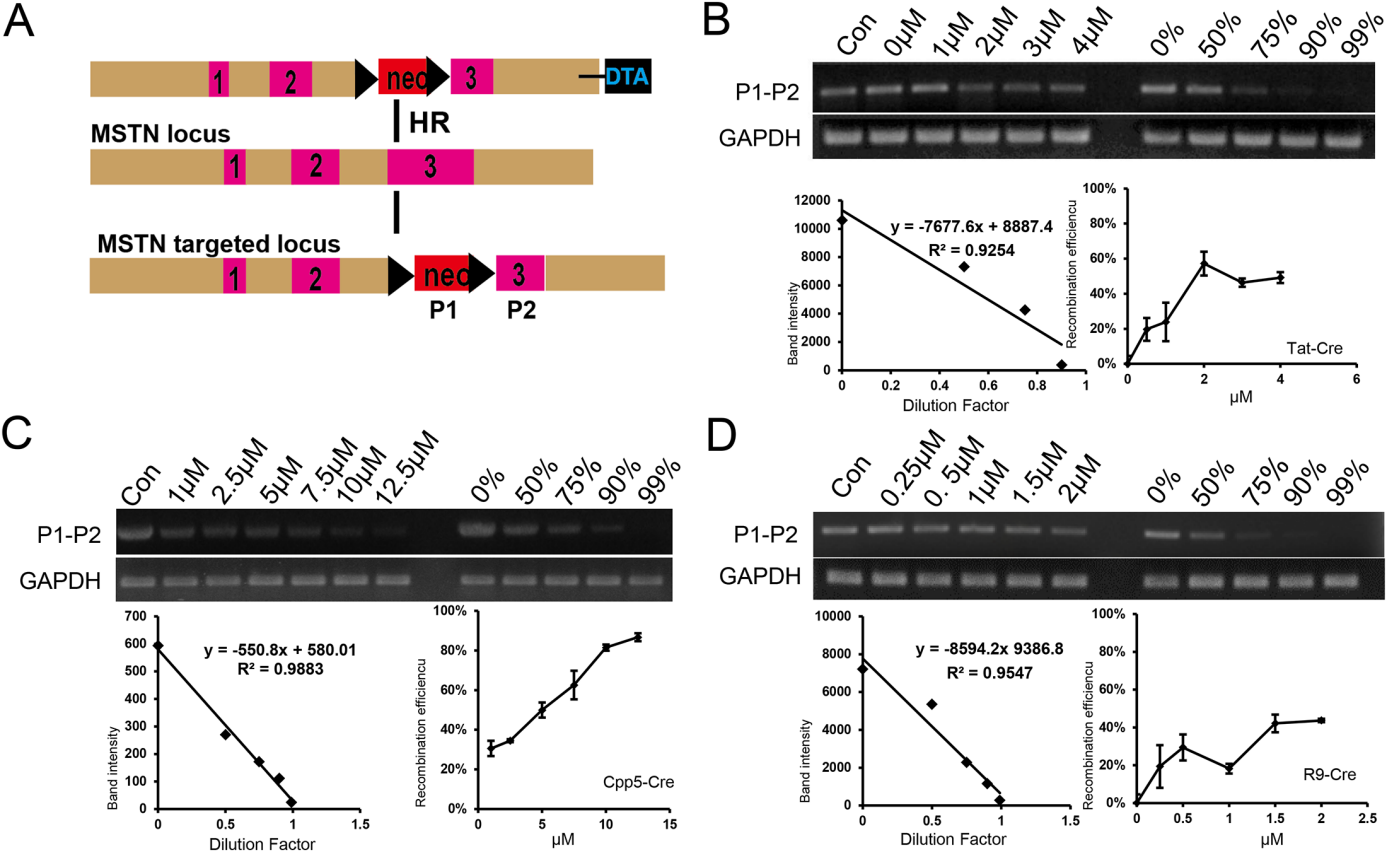
CPP–Cre mediated recombination efficiencies in porcine fetal fibroblasts (PFFs). Structure of the recombination substrate in loxP-Neo-loxP porcine fetal fibroblasts. The loxP-Neo-loxP PFFs, which were generated from the heterozygous *MSTN*^*+/−*^knockout pig generated previously in our laboratory, contains a single copy of Neo flanked by loxP sites, such that Cre-mediated recombination would remove Neo and the 600 bp fragment cannot be amplified using primers P1 and P2.(B-D) A standard curve for estimating the CPP–Cre recombination frequencies (39).Genomic DNA isolated from cells treated with CPP-Cre were serially diluted in a buffer and subjected to PCR analysis in a reaction volume of 25 μL. A standard curve for the estimation of deletion frequencies was generated. A plasmid containing the sequence of the PCR product corresponding to the genomic recombination induced by Cre was serially diluted in a solution containing genomic DNA isolated from pig fibroblast cells and the diluted samples were subjected to PCR analysis. The intensities of DNA bands corresponding to the recombination event were measured and plotted against the dilution factors. At high values, the band intensities reached a plateau and thus were excluded when the standard curve was plotted. Error bars, s.d. (n = 3).

To evaluate the ability of the three CPP**–**Cre fusion proteins to penetrate PFFs and subsequently perform recombination of a loxP-flanked substrate, a PFF line with a myostatin heterozygous knockout (*MSTN*^+/−^) containing a loxP-flanked neomycin-resistance gene (*Neo*) was used (Fig 4A). The two loxP-flanked *Neo* transgenic pigs were generated from the heterozygous *MSTN*^+/−^pigs generated in our laboratory. If the CPP**–**Cre fusion proteins invaded the PFFs and subsequently excised *Neo*, the 600 bp fragment could not be amplified using the primers P1 and P2 (Fig 4A). The standard curve method was used to estimate the recombination frequencies [43]. The *MSTN*^+/−^PFFs were incubated in media containing different concentrations of TAT**–**Cre, R9**–**Cre, and CPP5**–**Cre for 3 h. R9-Cre and TAT-Cre reached a maximum recombination efficiency of approximately 40% and 55%, respectively, at a concentration of 2 μM (Fig 4B and 4C). However, the recombination frequency of CPP5**–**Cre increased with the increasing protein concentration and reached nearly 90% at a concentration of 12.5 μM, which was the highest among the three CPPs (Fig 4D). We also using the P3 and P4 primer to perform this assay (S3 Fig). All the three CPP**–**Cre fusion proteins were able to induce recombination, suggesting that the CPPs were able to deliver the cargo protein to the porcine fetal fibroblasts and perform cargo protein function, but they exhibited different patterns.

### Both HA2 and chloroquine can improve the TAT–Cre recombination efficiency in PFFs with little or no toxicity

**Fig 5:**
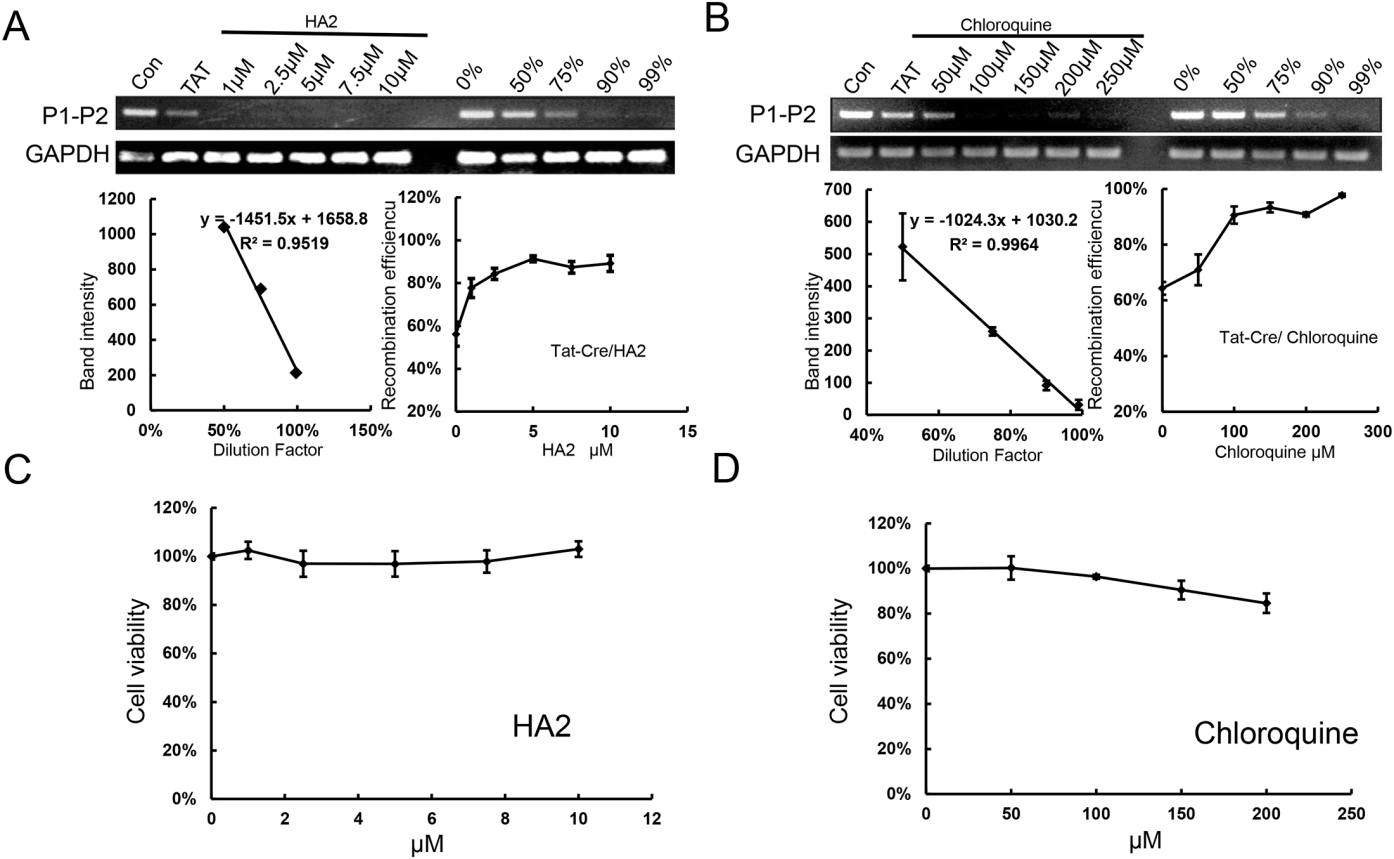
Effects of Lysosomotropic agents on TAT–Cre recombination efficiency and toxicity. Effects of different concentrations of HA2 (A) and chloroquine (B) on TAT**–**Cre-mediated recombination of a loxP-*Neo*-loxP target, using the standard curve method (39). Percentage of living cells (as an indication of potential toxicity) in porcine fetal fibroblasts treated with different concentrations of HA2 (C) and chloroquine (D). Error bars, s.d. (n = 3).

The above results showed that the TAT**–**Cre transduction efficiency was higher than the recombination efficiency and that TAT**–**Cre was localized to intracellular endocytic vesicles in PFFs (Fig 3D). Thus, we speculated that enhancing the escape from the endosome could increase the recombination efficiency of TAT**–**Cre in porcine fetal fibroblasts. We chose two small molecules for enhancing the endosome escape: HA2 and chloroquine, which reportedly can promote TAT**–**Cre release from the macropinosomes [44,45]. The PFFs were incubated for 3 h in 2 μM TAT**–**Cre medium supplemented with different concentrations of the two small molecules. Both 2.5 μM HA2 and 250 μM chloroquine could increase the recombination efficiency of TAT**–**Cre to nearly 90% (Fig 5A and 5B). The cytotoxicity of HA2 and chloroquine were assessed using the MTT assay on PFFs that have been cultured with various concentrations of the small molecules for 3 h. HA2 did not show significant cytotoxic effects even at a concentration of 10 μM in the cell culture medium. However, in PFFs, chloroquine was cytotoxic at a concentration 100 μM, at which about 5% of the cells were dead. These observations indicate that 5 μM HA2 significantly enhanced the efficiency of TAT**–**Cre recombination by enhancing the endosome escape without significant cytotoxic effect.

### Generating marker-free live transgenic pigs using TAT–Cre and CPP5–Cre

**Fig 6:**
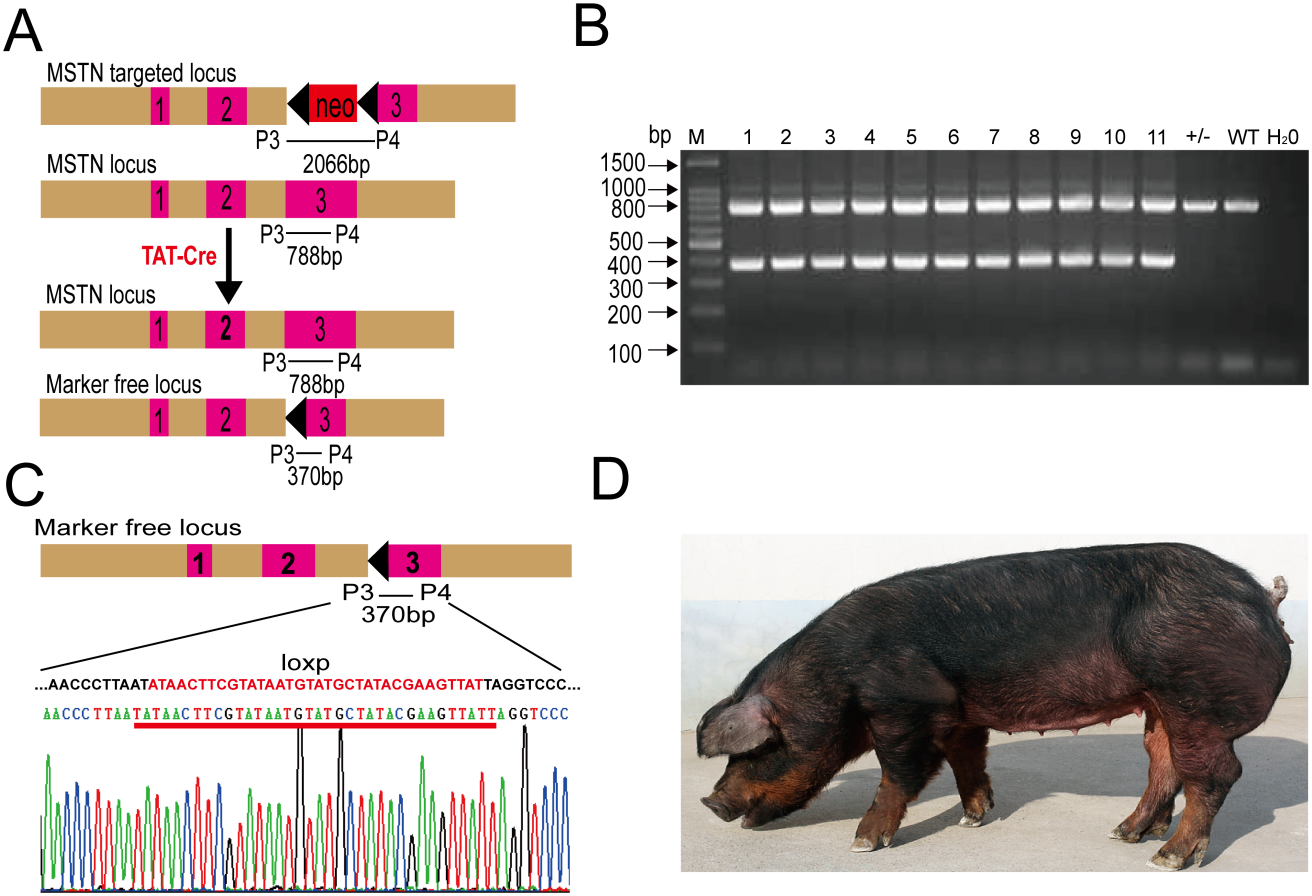
Generation of marker-free Monoallelic *MSTN* gene knockout pigs using TAT–Cre. (A) PCR-based genotyping scheme to differentiate the three *MSTN* alleles: WT, *MSTN*^+/−^with the *Neo*, and marker-free *MSTN*^+/−^. P3 and P4 primers designed to amplify 788-bp and 370-bp fragments from marker-free pigs. The 788-bp fragment could only be amplified from WT and *MSTN*^+/-^ pigs with a 30-s extension time. (B) Identification of marker-free *MSTN*^+/−^piglets using genomic PCR method. WT, wild-type; H_2_O, negative control; M^+/-^, *MSTN*^+/−^piglets containing *Neo*; 1**–**12 are marker-free *MSTN*^+/−^piglets. (C) PCR sequencing analysis of the marker free *MSTN*^+/−^piglets. The 370-bp product is a specific fragment for the marker-free pig genome including one loxP site between P3 and P4. (D) A live marker-free *MSTN*^+/−^pig.

Based on the above results, we speculated that CPP**–**Cre could remove the selective marker-gene in transgenic PFFs, which could subsequently be used to generate marker-free transgenic pigs. As a proof-of-principle experiment, we incubated *MSTN*^+/−^PFFs with TAT**–**Cre protein to remove *Neo* in the cell genome (S4 Fig), and then used the marker-free cells as nucleus donors. We obtained 12 piglets, which we used to confirm that the marker gene had been removed by PCR. The PCR was able to distinguish between the three potential *MSTN* alleles: wild-type, *Neo*, and loxP (Fig 6A and 6B). The PCR analysis of piglet tail DNA samples with P3 and P4 primers showed that both the 788-bp and 370-bp fragments could be amplified from the marker-free pigs. The 788-bp fragment could only be amplified from the WT and *MSTN*^+/-^ pigs using a 30 s extension time (Fig 6B), and the 370-bp PCR fragments were sequenced to further confirm the removal of the marker (Fig 6C). Currently, the marker free *MSTN*^+/−^pigs are growing normally (Fig 6D). Moreover, we also obtained marker free transgenic pigs using CPP5**–**Cre protein from BAC (bacterial artificial chromosome) transgenic pigs (S5 Fig), which carry *Neo* (pBAC-hLF-hLZ-Neo) and were generated in our lab previously. These results show that both TAT**–**Cre and CPP5**–**Cre can remove the marker gene in different transgenic pigs.

## Discussion

CPPs are one of the most promising tools for delivering various biologically active molecules into mammalian and plant cells both *in vitro* and *in vivo* [46–50]. They play key roles in basic and biomedical research [51–53]. However, there are very few studies on the application of CPPs in large animals [37,54], particularly pigs. To our knowledge, this study is the first to show that three cell-penetrating peptides, TAT, R9, and CPP5 (KLVPM), can deliver the Cre recombinase protein into fetal porcine fibroblasts and subsequently perform recombination with no apparent toxicity. Consistent with previous observations in HeLa or CHO cells [55–57], the cellular uptake mechanism of the three CPPs involved energy-dependent endocytosis; however, we did not study the transduction mechanism of the three CPPs in detail or determine whether it involves specific endocytic pathway or coexists with direct membrane translocation [58].

We found that all the three cell-penetrating peptides promoted transduction of the Cre protein in a concentration-dependent manner, and the transduction efficiencies of all the three proteins reached over 90% at higher concentrations. The CPP5 (KLVPM)**–**Cre fusion protein could be transduced into fetal porcine fibroblasts and could induce recombination with nearly 90% efficiency at 12.5 μM, but the maximum recombination efficiencies of TAT**–**Cre and R9**–**Cre were 55% and 45%, respectively. The data presented here suggested that the rate-limiting step of TAT**–**Cre and R9**–**Cre-mediated recombination is the endosome escape, but higher concentrations CPP5**–**Cre is not as limited. Interestingly, all the three fluorescently labeled fusion Cre recombinase proteins at 2 μM localized to the intracellular endocytic vesicles in the live cells, and both HA2 and chloroquine improved the recombination efficiency of TAT**–**Cre. These results indicate that the cellular uptake mechanisms of the three CPPs in pig primary cells are different, that the uptake mechanisms of TAT**–**Cre and R9**–**Cre might involve the endocytosis pathway, and that the uptake mechanism of CPP5**–**Cre protein might also involve other pathways, including direct membrane translocation, clathrin-and caveolin-mediated endocytosis, macropinocytosis, or nonclathrin-noncaveolae-dependent endocytosis [59,60]. The recombination efficiency of TAT**–**Cre in hES cells was reported to be nearly 100% [22], but it was only 55% in pig primary cells, which indicates that the recombination efficiency of CPP**–**Cre is cell type-dependent and concentration-dependent, which is consistent with the results of previous studies [61,62].

The removal of antibiotic-selectable marker genes is a challenging problem for transgenic animal research, especially in pigs. Although marker-free genetically modified pigs can be obtained by pronuclear microinjection (PNI) [63], which has a very low transgenic efficiency and requires extensive waiting period for establishment of the transgenic animal lines, especially in the case of pigs. Recently, two groups have efficiently generated transgenic pigs by cytoplasmic injection of the Sleeping Beauty systems [64,65]. These studies provide strong technological support for the production of marker-free transgenic pigs.

It has been reported that marker-free pigs can be obtained using the Sleeping Beauty transposon system [66], but this method is labor-intensive and requires screening for integration of a single-copy marker gene and breeding. The marker gene can also be removed by introduction of Cre-recombinase into target cells, which is currently achieved by transfection and expression of plasmid DNA or mRNA or by virus-mediated transduction. However, the efficiency of non-viral DNA transfection is consistently very low in many cell types, and the use of viral vectors for transduction, although more efficient, involves a complex and laborious manipulation associated with safety issues.

In this study, we established a safe, simple, and efficient method to remove the marker gene in transgenic pigs using the CPP**–**Cre protein. This method has many advantages. Firstly, it is very safe because no other foreign nucleic acids are introduced, unlike in previous approaches in cattle and mice that used plasmid DNA to express the Cre protein [67,68], and constitutive expression of Cre has been shown to be toxic in several cell types [69,70]. Secondly, this technique is simple and easy to perform, involving only the addition of fusion Cre recombinant proteins into the medium during cell culture. Thirdly, it is highly efficient; the transduction efficiency of the three CPPs was over 90%, and the recombination efficiencies of TAT**–**Cre and R9**–**Cre obtained were 55% and 45%, respectively. Moreover, the recombination efficiency of CPP5**–**Cre reached 90% at high concentrations. Because the purification of R9**–**Cre was difficult, the TAT**–**Cre and CPP5**–**Cre were used to obtain marker-free *MSTN*^+/−^pigs. We also obtained marker-free live *MSTN*^+/−^pigs using TAT**–**Cre and CPP5**–**Cre-treated *MSTN*^+/−^PFFs as nucleus donors in nuclear transfer experiments. The pigs are growing normally and breeding the next generation, which provides evidence that there was no severe damage to the host cells during the process of protein transduction.

In summary, we expanded the CPP technology to transgenic pig research field and established a simple, efficient, and cost-effective method to remove the marker gene in transgenic pigs using CPP**–**Cre proteins. We expect that this technology can be applied in a variety of experimental protocols, many of which can be evaluated *in vitro* or *in vivo* in pig models. This technology provides an important basis for modifying livestock genomes, which are important for both biomedical and agricultural applications.

## Conclusions

In this study, we report that three CPPs (CPP5, TAT, and R9) can efficiently deliver active Cre recombinase protein into PFFs via an energy-dependent endocytosis pathway. Moreover, we found that 12.5 μM CPP5**–**Cre protein could invade pig primary cells and subsequently perform recombination with almost 90% efficiency. HA2 and chloroquine could improve the recombination efficiency of the TAT**–**Cre. We suggest that the effect of the CPPs is type-dependent and concentration-dependent. The normal live marker-free genetically modified pigs were also obtained using TAT**–**Cre and CPP5**–**Cre. All these results suggest that the application of the CPPs for generation of transgenic pigs is feasible.

## Supporting information

S1 Table Summary of the primers. (DOCX)

S1 Fig FACS analysis of TAT mediated transduction efficiency in pig fibroblast cells. Purified Cre and TAT-Cre protein is labeled with Alexa 488 fluorescent group, and diluted to 2 μM with DMEM. Pig fibroblast cells are incubated with Alexa 488 Cre or TAT-Cre for 2 hours in 37°C, then washing these cells with heparin solution and trypsinizing them for FACS sorting analysis. A. FACS sorting gate is sent by cells treated with DMEM solution; this FACS histogram shows negative (non-fluorescent) events. B-C FACS sorting analysis of Alexa 488 labeled Cre and TAT-Cre treated pig fibroblast cells. D. Statistical analysis of Alexa 488 positive cells percentage (n = 3, p<0.01).
(TIF)

S2 Fig Sorting profile for the Alexa 488 labeled PTD-Cre treated pig fibroblast cells. After purification, PTD-Cre protein is dissolved in PBS, and conjuncted with fluorescent group by Alexa Fluor 488 Protein Labeling Kit (Molecular Probe, A10235). Pig fibroblast cells is incubated with Alexa 488 labeled PTD-Cre protein for 2 hours in 37°C, then cells are washed by heparin solution and trypsinized for FACS sorting analysis. A-C.Histograms of flow cytometry for cultured pig fibroblast cells. The negative/positive gate is determined using vehicle control (PBS and DMEM) treated PFFs (left figure). After treatment of Alexa 488 PTD-Cre, pig fibroblast cells show significant distribution in Alexa 488 positive group.
(JPG)

S3 Fig CPPs–Cre mediated recombination efficiencies in porcine fetal fibroblasts (PFFs). (A) Structure of the recombination substrate in loxP-Neo-loxP porcine fetal fibroblasts. The loxP-Neo-loxP PFFs, which were generated from the heterozygous *MSTN*^+/−^knockout pig that our laboratory previously produced, contains a single copy of a Neo flanked by loxP sites such that Cre-mediated recombination would remove the Neo, therefore a 370 bp fragment from marker-free allele and a 788 bp fragment from WT allele can be amplified using primers P3 and P4. (B-D) A standard curve for estimating the CPPs-Cre recombination frequencies. Genomic DNA isolated from cells treated with CPP-Cre were serially diluted in a buffer and subjected to PCR analysis in a reaction volume of 25 μL. Standard samples for the estimation of deletion frequencies. A plasmid containing a PCR product corresponding to the genomic recombination induced by Cre was serially diluted in a solution containing genomic DNA isolated from WT pig fibroblast cells and the diluted samples were subjected to PCR analysis. Intensities of DNA bands corresponding to the recombination event were showed and plotted against dilution factors.
(TIF)

S4 Fig Excision of selectable gene from *MSTN*^+/-^ PFFs by TAT-Cre. (A) Diagrams of obtain maker free *MSTN*^*+/-*^ PFFs by TAT-Cre mediated method. The primordial *MSTN*^*+/-*^ PFFs genome include *MSTN* targeted locus and WT locus. The 1102 bp and 664 bp fragments can be amplified in 30 s extension time in marker free *MSTN*^*+/-*^ PFFs, using primer M1 and M2. (B) PCR analysis of the marker free *MSTN*^*+/-*^ PFFs clones treated by TAT-Cre. Twenty cell clones were identified by genomic PCR, the positive clones can amplify 1102 bp and 664 bp fragment in 30 s extension time.
(TIF)

S5 Fig Generate marker free live pig using the CPP5-Cre. (A) Structure of identify the un-marker free and marker free hLZ-BAC transgenic pigs. The 509 bp fragments can be amplified from the marker free transgenic pigs using P5 and P6 primers, and the 2306 bp fragments could be amplified from the un-marker free transgenic pigs. (B) Identification of marker free hLZ-BAC transgenic piglets by genomic PCR. 1–4 are marker free transgenic piglets; H2O, Negative control; B-N, Un-marker free hLZ-BAC transgenic piglets. (C) PCR sequencing analysis of the four-marker free hLZ-BAC transgenic piglets. The 509 bp marker free fragment including one loxP site between the P5 and P6 sequence. (D) The live marker free hLZ-BAC transgenic pig. Fig 2d was taken by Z.S. and Q.K.
(TIF)
