# Supplementary material for: Cell-penetrating peptide-driven Cre recombination in porcine primary cells and generation of marker-free pigs
Source: PLoS One. 2018 Jan 9;13(1):e0190690. doi: 10.1371/journal.pone.0190690 (PMC5760039; doi:10.1371/journal.pone.0190690)
Supplement: S1 Table — (DOCX) [file pone.0190690.s001.docx]

**S1 Table. Summary of the primers.**

| Name | Sequence |
| --- | --- |
| CPP5-R | CATGGGCAAACTGCCGGTTATGGGCCA |
| CPP5-F | TATGGCCCATAACCGGCAGTTTGCC |
| R9-R | CATGGGCCGTCGTCGTCGTCGTCGTCGTCGTCGTGGCCA |
| R9-F | TATGGCCACGACGACGACGACGACGACGACGACGGCC |
| mCherry-F | AGCTTGATGGGGATCCGCCACCATGGTGAGCAAGGGCGA |
| mCherry-R | GCCCAAGCTTTCGCGAGCTCGCTTAAGATACA |
| Neo-F | AGCTTGGGCTTGAACATCGAGCG |
| Neo-R | TCACCATGGTGGCGTATAACTTCGTATAATGTATGCTATACGAAGTTATGGATCTGTCGATCGACGGATCGA |
| EGFP-F | ACGCCACCATGGTGAGCAAGG |
| EGFP-R | TCGAGGCTGATCAGCGAGCTCTTACTTGTACAGCTCGTCCATGC |
| M-1 | GAGCATACCTACCCATCAGAC |
| M-2 | TAATTCACCAGAACACAAGGAG |
| P1 | CGACTGTGCCTTCTAGTTGCC |
| P2 | CATGAGCACCCACAGCGATCT |
| P3 | ACTTTCCCATTACAGTCCCTA |
| P4 | CATCTTTGTGGGAGTACAGCA |
| P5 | GATTAGCTTTGTAAATTTGTG |
| P6 | TTGGTGTTGTAGTTACCCT |
