# Supplementary figures and images for: Cell-penetrating peptide-driven Cre recombination in porcine primary cells and generation of marker-free pigs
Source: PLoS One. 2018 Jan 9;13(1):e0190690. doi: 10.1371/journal.pone.0190690 (PMC5760039; doi:10.1371/journal.pone.0190690)

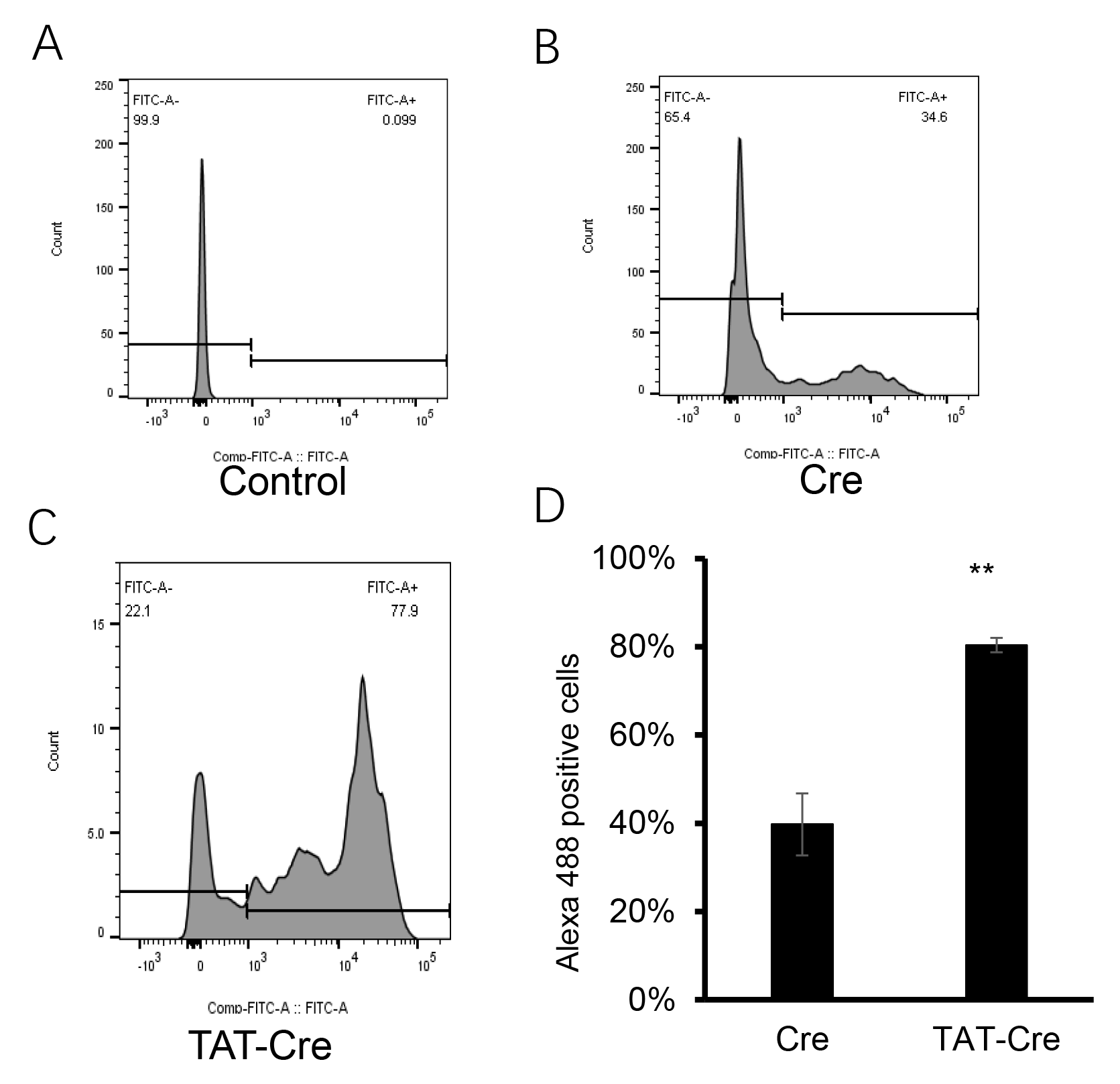

Supplement: S1 Fig — Purified Cre and TAT-Cre protein is labeled with Alexa 488 fluorescent group, and diluted to 2 μM with DMEM. Pig fibroblast cells are incubated with Alexa 488 Cre or TAT-Cre for 2 hours in 37°C, then washing these cells with heparin solution and trypsinizing them for FACS sorting analysis. A. FACS sorting gate is sent by cells treated with DMEM solution; this FACS histogram shows negative (non-fluorescent) events. B-C FACS sorting analysis of Alexa 488 labeled Cre and TAT-Cre treated pig fibroblast cells. D. Statistical analysis of Alexa 488 positive cells percentage (n = 3, p<0.01). (TIF) [file pone.0190690.s002.tif]

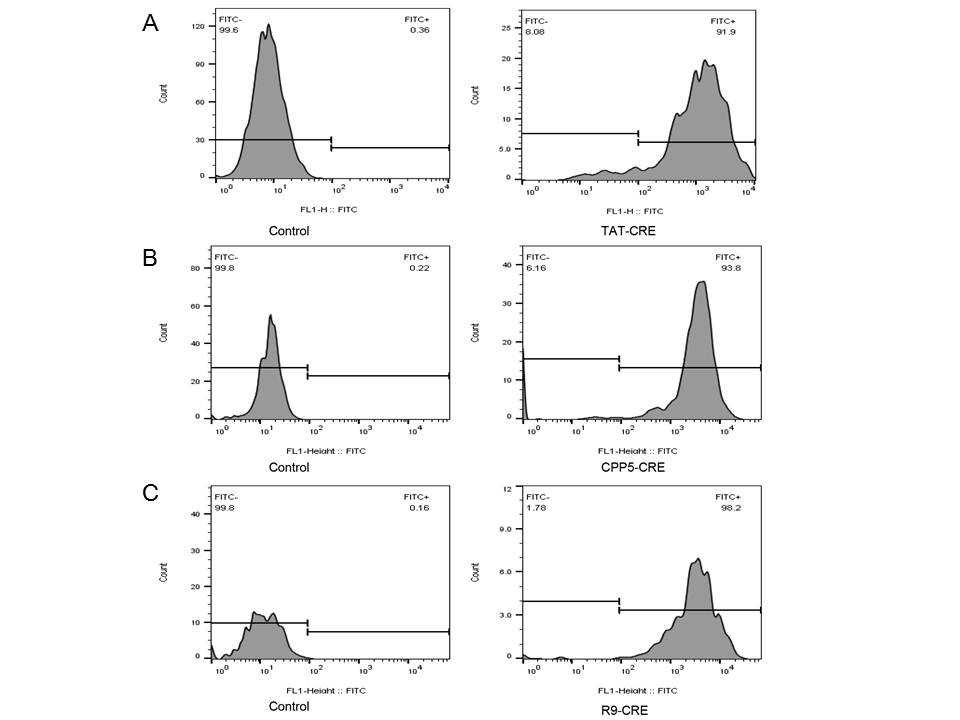

Supplement: S2 Fig — After purification, PTD-Cre protein is dissolved in PBS, and conjuncted with fluorescent group by Alexa Fluor 488 Protein Labeling Kit (Molecular Probe, A10235). Pig fibroblast cells is incubated with Alexa 488 labeled PTD-Cre protein for 2 hours in 37°C, then cells are washed by heparin solution and trypsinized for FACS sorting analysis. A-C.Histograms of flow cytometry for cultured pig fibroblast cells. The negative/positive gate is determined using vehicle control (PBS and DMEM) treated PFFs (left figure). After treatment of Alexa 488 PTD-Cre, pig fibroblast cells show significant distribution in Alexa 488 positive group. (JPG) [file pone.0190690.s003.jpg]

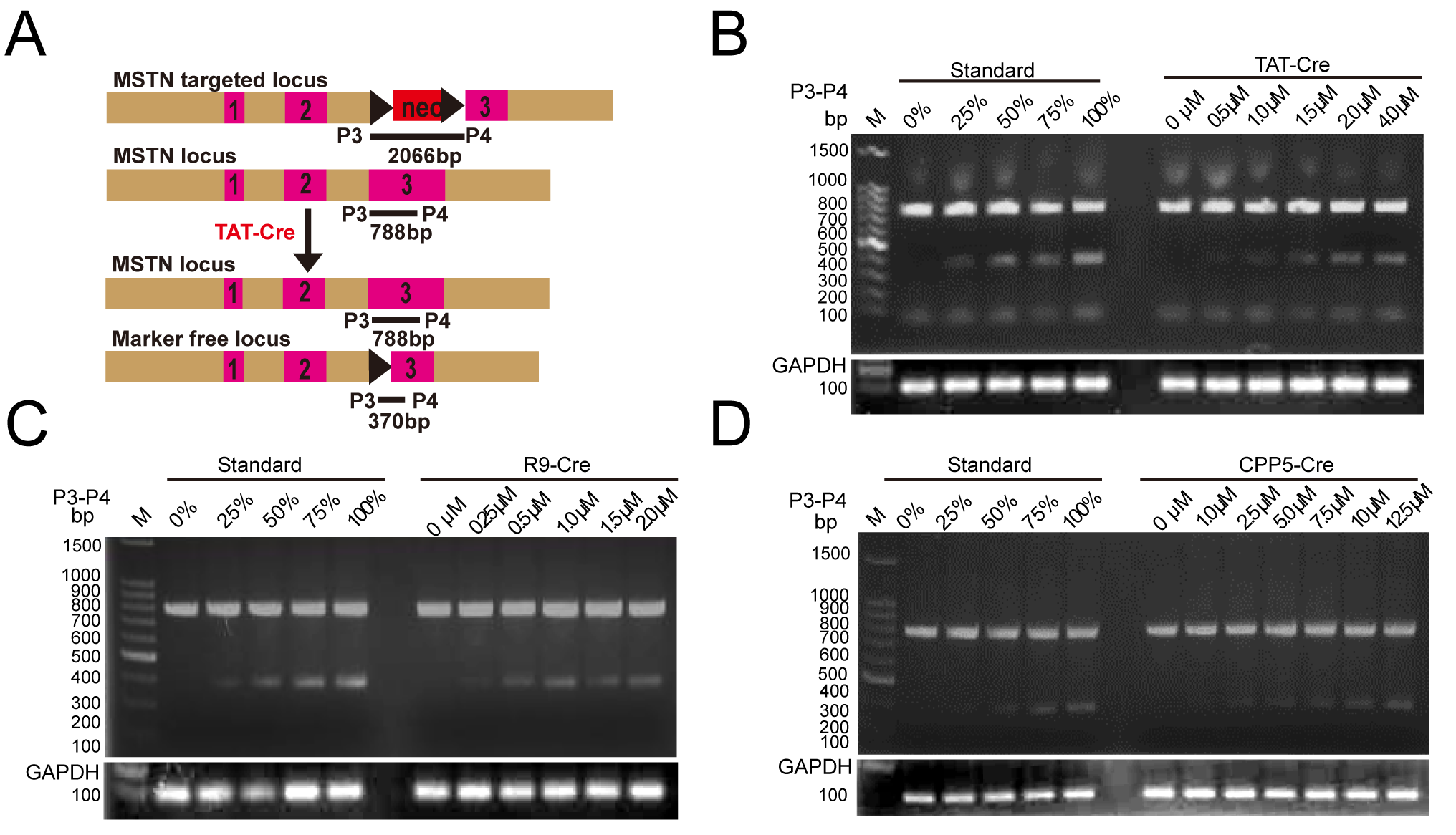

Supplement: S3 Fig — (A) Structure of the recombination substrate in loxP-Neo-loxP porcine fetal fibroblasts. The loxP-Neo-loxP PFFs, which were generated from the heterozygous MSTN+/−knockout pig that our laboratory previously produced, contains a single copy of a Neo flanked by loxP sites such that Cre-mediated recombination would remove the Neo, therefore a 370 bp fragment from marker-free allele and a 788 bp fragment from WT allele can be amplified using primers P3 and P4. (B-D) A standard curve for estimating the CPPs-Cre recombination frequencies. Genomic DNA isolated from cells treated with CPP-Cre were serially diluted in a buffer and subjected to PCR analysis in a reaction volume of 25 μL. Standard samples for the estimation of deletion frequencies. A plasmid containing a PCR product corresponding to the genomic recombination induced by Cre was serially diluted in a solution containing genomic DNA isolated from WT pig fibroblast cells and the diluted samples were subjected to PCR analysis. Intensities of DNA bands corresponding to the recombination event were showed and plotted against dilution factors. (TIF) [file pone.0190690.s004.tif]

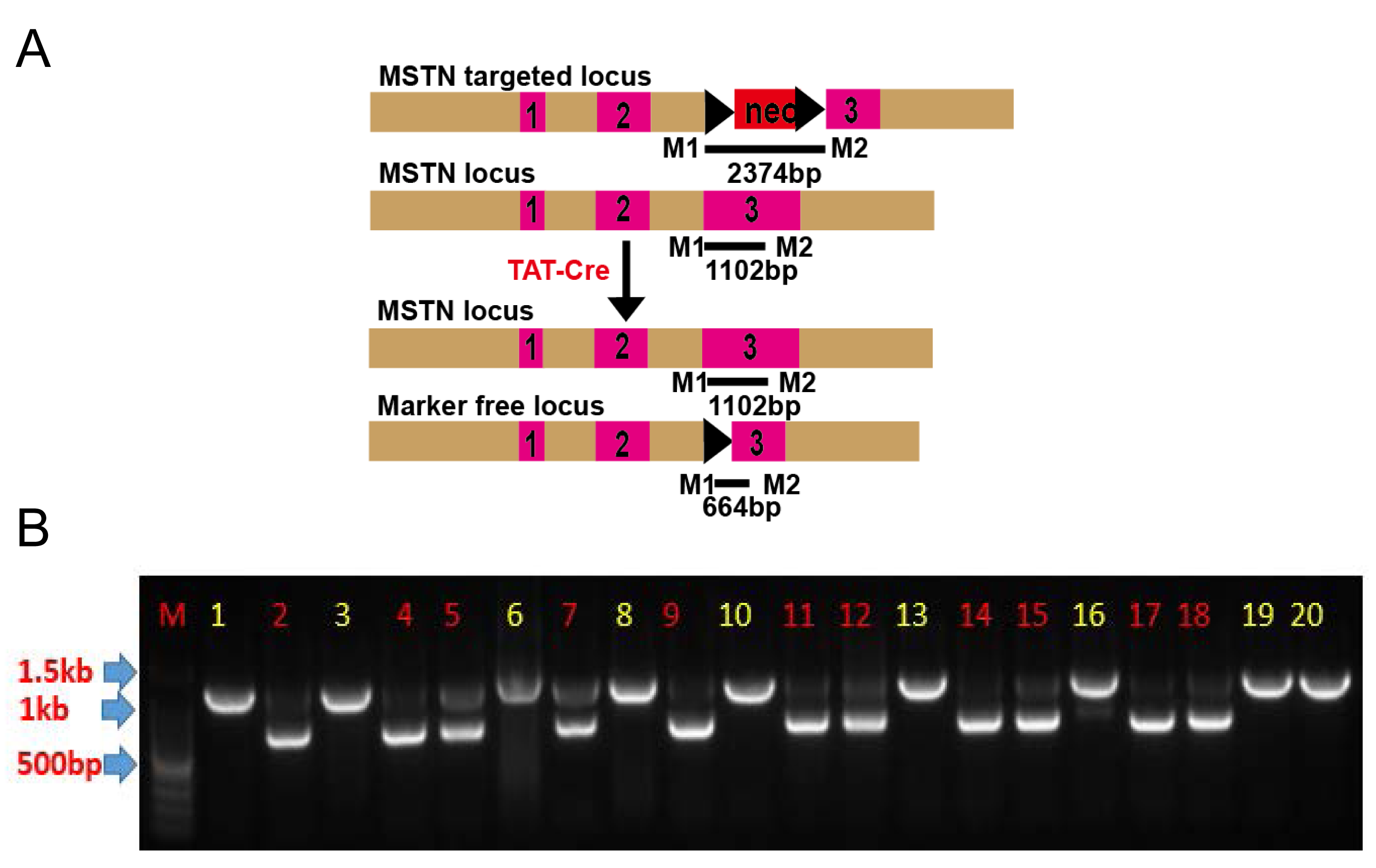

Supplement: S4 Fig — (A) Diagrams of obtain maker free MSTN+/- PFFs by TAT-Cre mediated method. The primordial MSTN+/- PFFs genome include MSTN targeted locus and WT locus. The 1102 bp and 664 bp fragments can be amplified in 30 s extension time in marker free MSTN+/- PFFs, using primer M1 and M2. (B) PCR analysis of the marker free MSTN+/- PFFs clones treated by TAT-Cre. Twenty cell clones were identified by genomic PCR, the positive clones can amplify 1102 bp and 664 bp fragment in 30 s extension time. (TIF) [file pone.0190690.s005.tif]

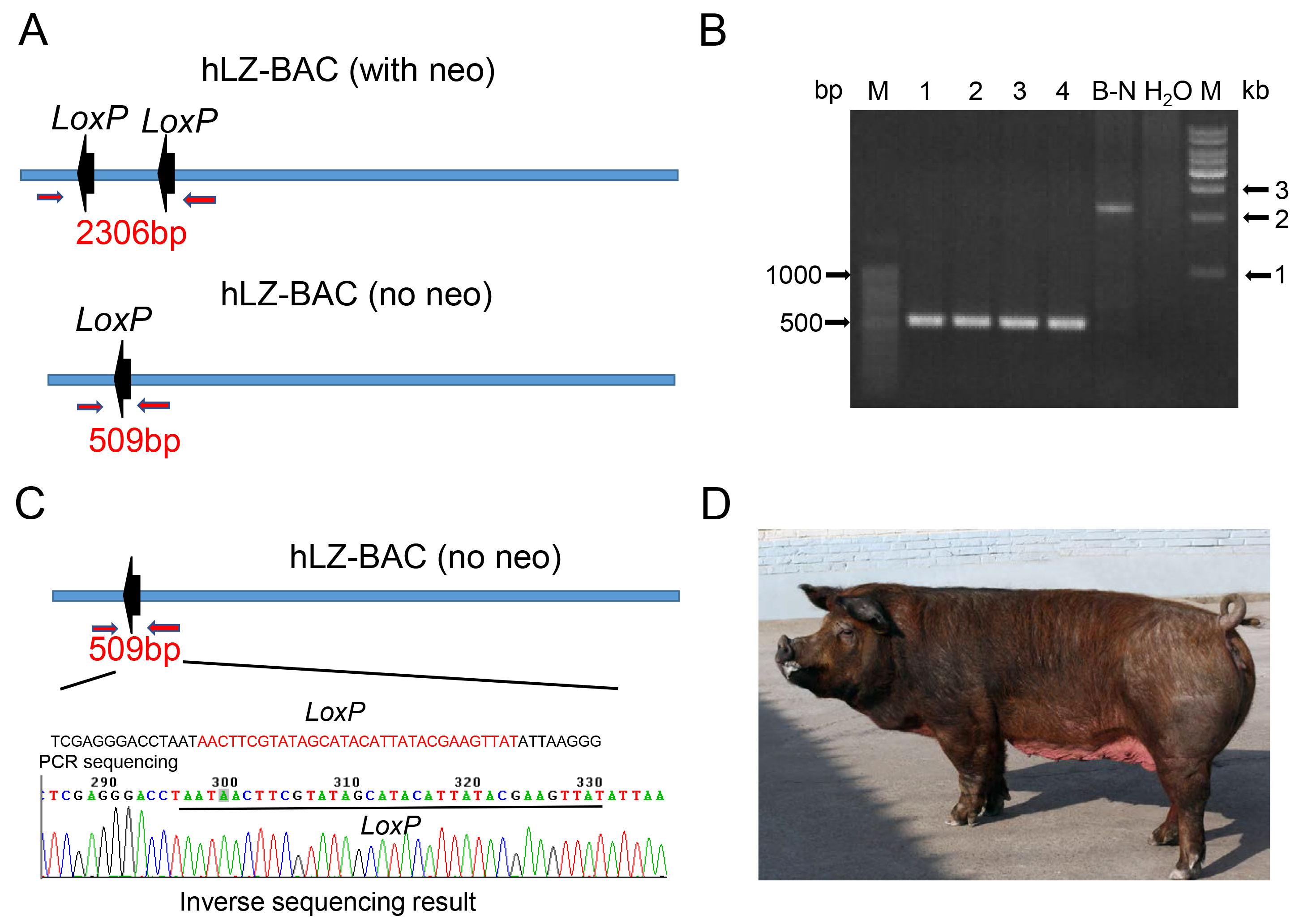

Supplement: S5 Fig — (A) Structure of identify the un-marker free and marker free hLZ-BAC transgenic pigs. The 509 bp fragments can be amplified from the marker free transgenic pigs using P5 and P6 primers, and the 2306 bp fragments could be amplified from the un-marker free transgenic pigs. (B) Identification of marker free hLZ-BAC transgenic piglets by genomic PCR. 1–4 are marker free transgenic piglets; H2O, Negative control; B-N, Un-marker free hLZ-BAC transgenic piglets. (C) PCR sequencing analysis of the four-marker free hLZ-BAC transgenic piglets. The 509 bp marker free fragment including one loxP site between the P5 and P6 sequence. (D) The live marker free hLZ-BAC transgenic pig. Fig 2d was taken by Z.S. and Q.K. (TIF) [file pone.0190690.s006.tif]
